# Preparation of Cement Composites with Ordered Microstructures via Doping with Graphene Oxide Nanosheets and an Investigation of Their Strength and Durability

**DOI:** 10.3390/ma9110924

**Published:** 2016-11-14

**Authors:** Shenghua Lv, Jia Zhang, Linlin Zhu, Chunmao Jia

**Affiliations:** 1 College of Bioresources Chemical and Materials Engineering, Shaanxi University of Science and Technology, Xi’an 710021, China; zhull@yahoo.com; 2 College of Environment Science and Engineering, Shaanxi University of Science and Technology, Xi’an 710021, China; zhangjia201611@yahoo.com; 3 College of Chemistry and Chemical Engineering, Shaanxi University of Science and Technology, Xi’an 710021, China; jiacm1205@yahoo.com

**Keywords:** graphene oxide nanosheets, cement composites, microstructure, reinforcement, durability

## Abstract

The main problem with cement composites is that they have structural defects, including cracks, holes, and a disordered morphology, which significantly affects their strength and durability. Therefore, the construction of cement composites with defect-free structures and high strength and long durability is an important research topic. Here, by controlling the size and chemical groups of graphene oxide nanosheets (GONs) used for doping, we were able to control the entire cement matrix to form an ordered microstructure consisting of polyhedron-like crystals and exhibit flower-like patterns. The cracks and holes in the cement matrix just about vanished. The compressive and flexural strengths as well as the parameters for the durability assessment of the corresponding cement composites obviously improved compared with the control samples. Thus, the formation mechanism of the cement matrix with the ordered microstructure is proposed, and a proper explanation is given to regulation action.

## 1. Introduction

The mechanical properties and durability of a material are mostly dependent on its microstructure [1,2,3]. Therefore, it is very important to study the preparation methods of materials with ideal microstructures. Currently, for commonly used cement composites such as concrete and mortar, the main problems are that it always has a disordered microstructure with many cracks and holes, which results in the deterioration of their performance and a reduction in their durability [4,5,6,7,8]. These problems are closely associated with the shape and aggregate state of cement hydration products [9,10,11]. Generally, cement hydration products always have irregular shapes and randomly agglomerate, resulting in the formation of disordered microstructures [12,13,14]. The main solution for reducing the cracks of a cement matrix still mainly depends on filling various fibers and nano mineral materials [15,16,17]. These fibers and materials cannot control the shape and aggregate state of cement hydration products; the irregular agglomeration of the cement hydration crystals with an irregular shape inevitably produces many cracks and holes in the cement matrix. Therefore, an ability to control the formation of regular cement hydration products and ordered microstructures can solve these problems. However, there has been limited research in this field so far. The reason is that no methods or materials that can exert complete control on the shapes and ordered arrays of cement hydration products are available.

The emergence of graphene oxide (GO) has presented a new opportunity to resolve these problems [18,19,20]. Graphene oxide nanosheets (GONs) can be used to form defect-free microstructures with traditional materials such as polymers [21,22], metals [23], ceramics [24,25], and fibers [26] by its template and assemble effects to obtain a wide range of tunable properties for use in various applications [27,28,29,30]. These results have inspired us to solve the problems faced by cement composites. In previous studies, we first found that GONs can be used to regulate cement hydration products to form regular bar-like, flower-like, and polyhedron-like crystals and further condense into ordered microstructures [31,32], which can markedly reduce cracks and holes and significantly improve the strength and toughness of cement composites. We also found that it is hard to form large-scale ordered microstructures in entire cement composites [33,34]. Meanwhile, some researchers have also begun studying the effects of GONs on the mechanical properties of cement composites, but they have not focused on the use of GONs regulated cement hydration products and microstructures [35,36]. In this paper, we study the methods of forming an ordered microstructure in entire cement composites droping with GONs. Relations between the ordered microstructure and strength and durability are also investigated. The formation mechanism of the ordered microstructure of the cement matrix was elucidated according to its SEM morphologies.

## 2. Materials and Methods

### 2.1. Materials and Chemicals

Powdered graphite had an average diameter of 30 μm. Polycarboxylate superplasticizers (PCs, with a content of 40% and a water-reducing rate of 32%) were supplied by Xi’an Xiaoshenke Additive Co., Ltd. (Xi’an, China). Portland cement (P.O. 42.5) and standard sand were supplied by Shaanxi Tianhao concrete Co., Ltd. (Xi’an, China). The chemical composition of the cement is shown in Table 1. The main chemicals used were concentrated sulfuric acid (H_2_SO_4_, 98%), potassium permanganate (KMnO_4_), sodium nitrate (NaNO_3_), and hydrogen peroxide (H_2_O_2_, 30%). All chemicals used are of reagent purity and not any treatment.

### 2.2. Preparation of GONs

A three-necked flask was placed in an ice bath at 5 °C, and 3 g of graphite, 60 g of concentrated H_2_SO_4_, and 3 g of NaNO_3_ were added and mixed well. Then, 12 g of KMnO_4_ was slowly added to the flask over 30 min with stirring, then kept at 5 °C for 1 h and kept at 35 °C for 6 h. Then, 200 mL of deionized water was added and kept at 70 °C for 1 h, after which 30 g of H_2_O_2_ was dripped into the flask over 20 min. The final product was purified by centrifugation, precipitation, and washing repeatedly with deionized water until the pH of the washing water was 7.0. The graphite oxide was further treated with ultrasonic processing for 60 min.

### 2.3. Preparation of GON/Cement Composites

The GON/cement composites were prepared by uniformly mixing water, PCs, and GONs first, and then cement and sand via stirring. The weight ratio of the cement/water/PCs/GONs was 450:1350:160:0.9:0.09. The sample sizes were 40 mm × 40 mm × 160 mm and 100 mm × 100 mm × 400 mm, respectively. The specimens were removed from the mold after 24 h and cured in standard conations until testing.

### 2.4. Test Methods

The chemical groups in the GONs were measured by Fourier-transform infrared spectroscopy (FTIR; EQUINOX-55, Bruker, Ettlingen, Germany) and X-ray photoelectron spectroscopy (XPS; XSAM 800, Kratos, Manchester, UK). The microstructure and the size distribution of GONs were examined by atomic force microscopy (AFM; SPI3800N/SPA400, Seiko, Osaka, Japan) and a laser particle analyzer (NANO-ZS90, Zetasizer, Worcestershire, UK). X-ray diffraction (XRD; D/max2200PC, Rigaku, Osaka, Japan) was used to examine the crystalline. The microstructures of GON/cement composites were determined with a scanning electron microscope (SEM; S-4800, Hitachi, Tokyo, Japan). The elemental compositions were determined with an energy-dispersive X-ray spectrometer (EDS) (EDAX, Cassatt, SC, USA), which was coupled with the S-4800 SEM.

The compressive strength was tested with a concrete compressive strength tester (JES-300, Wuxi, China) at a pressure increase rate of 1 MPa/s. The flexural strength of the GON/cement composites was determined using a concrete three-point flexural strength tester (DKZ-500, Wuxi, China) at a pressure increase rate of 0.25 MPa/s. The water penetration, the freeze thawing, and the carbonation experiment were carried out by GB/T5082-2009 (National Standard of China).

## 3. Results and Discussion

### 3.1. Structural Characterization of GONs

**Figure 1:**
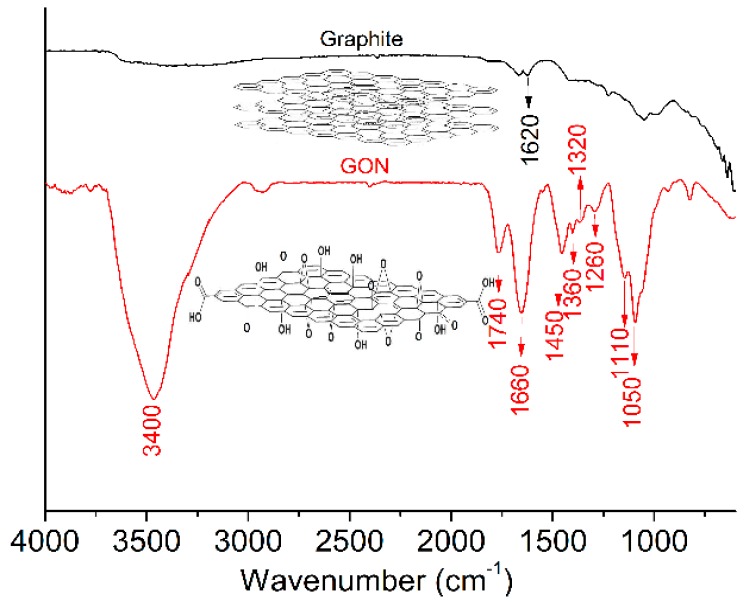
Fourier-transform infrared spectroscopy (FTIR) spectra of graphite (**black**) and graphene oxide nanosheets (GONs) (**red**).

**Figure 2:**
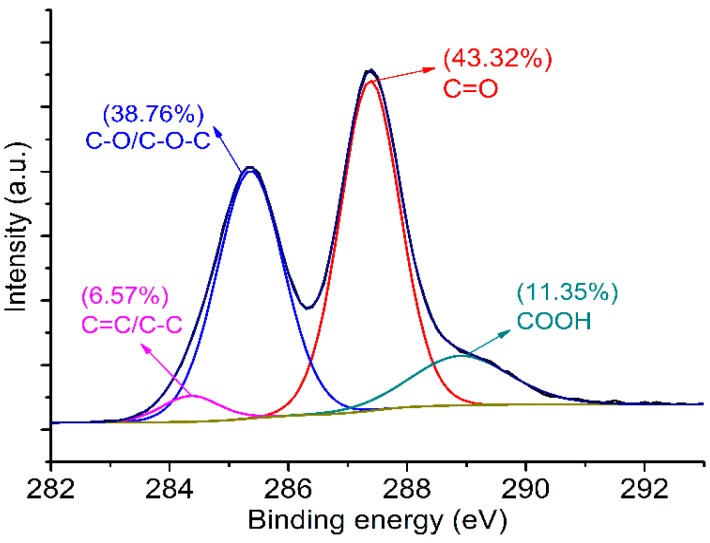
X-ray photoelectron spectroscopy (XPS) spectra of GONs.

The FTIR spectra of GONs and graphite are shown in Figure 1. The results indicate that the GONs contain hydroxyl groups (–OH, 3350 cm^−1^), carboxyl groups (COOH, 1740 cm^−1^), carbonyl groups (C=O, 1660 cm^−1^), and ether bonds (–C–O–C–, 1450, 1360, 1320, 1260, 1100, 1050 cm^−1^), which are not present on the FTIR spectra of graphite. The XPS spectra of GONs are shown in Figure 2, indicating that the carbon bonds in GONs were C=C, C–OH/C–O–C, C=O, and COOH in a proportion of 6.57%, 38.76%, 43.32%, and 11.35%, respectively. The results suggest that GONs contain hydroxyl, epoxy, carbonyl, and carboxyl groups compared with graphite.

**Figure 3:**
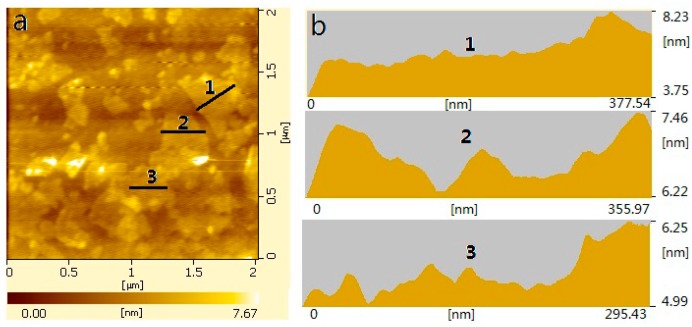
(**a**) Atomic force microscopy (AFM) images of GONs; (**b**) AFM surface patterns in the positions 1, 2, and 3 of Figure 3a.

**Figure 4:**
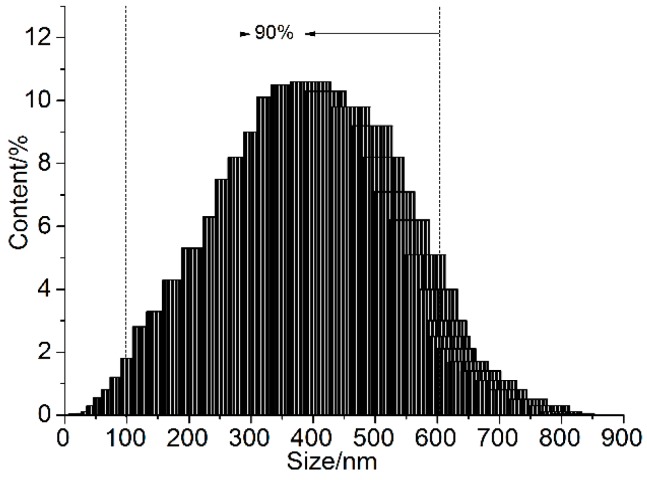
The size distribution of the GONs in an aqueous solution.

The AFM images of GONs are shown in Figure 3. The results reveal that the thickness of the GONs is less than 7.67 nm, and their length/width is in the range of 50–600 nm. The results also indicate that the surfaces of GONs are usually not flat and have a very rough surface. This may be attributed to the random overlap of many GONs. A laser particle analyzer was used to confirm the size distribution of the GONs, and the result is shown in Figure 4. The size distribution of the GONs was in the range of 10–800 nm, and 90% of the GONs were in the range of 100–600 nm. The results of the AFM images and the size distribution indicated that GONs are multi-layer nanosheets.

**Figure 5:**
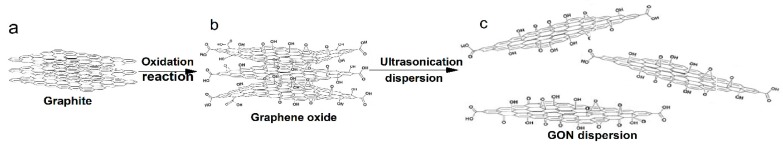
Schematic diagram of the preparation of GONs. (**a**) Compact aggregates of graphite; (**b**) Edge dilation state of oxided graphite in oxidied initiation; (**c**) Dispersed state of GONs.

Figure 5 shows a possible formation mechanism for GONs. Original graphite is comprised of compact aggregates of a flat sheet of carbon one atom thick, and it is hard to disperse into nanosheets (Figure 5a). When graphite is oxidized, the oxidizer can soak slowly into the lamellar structure of graphite and produce many hydrophilic chemical groups on its interfaces especially at its edges, which resulted in edge dilation (Figure 5b). The enlarged edges provide a pathway for oxidants to penetrate into the deeper level and make it easy dispersion, exfoliated in later ultrasonic processing (Figure 5c).

### 3.2. Microstructure of GON/Cement Composites

**Figure 6:**
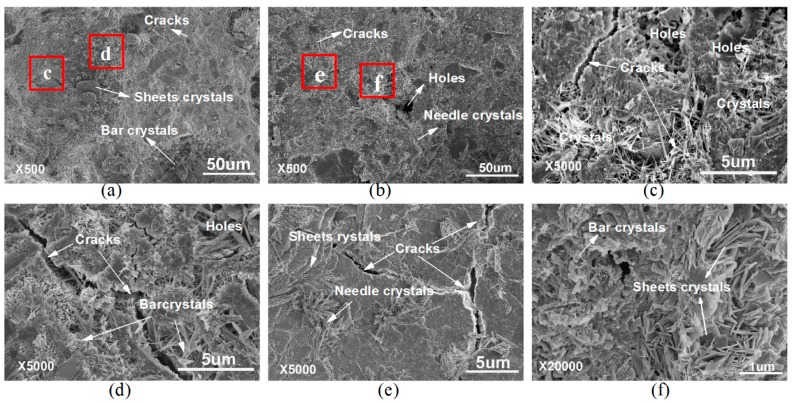
Scanning electron microscope (SEM) images of cement composites without GONs at 28 days with low magnification times (**a**,**b**) and high magnification times (**c**–**f**).

The effect of GONs on cement composites were investigated by comparing the microstructure and performances of the composites in both the absence and the presence of GONs. The microstructure of cement composites without GONs was first investigated by observing the SEM images of its fracture interfaces. The SEM images are shown in Figure 6. Figure 6a,b is the SEM images of the fracture interfaces magnified 500 times, showing that the whole cement matrix has an irregular microstructure. Figure 6c–f shows SEM images magnified 5000 times, showing that the structure characteristic of the cement matrix is irregular and disordered, and contains many holes and cracks, as well as some needle-like, bar-like, and sheet-like crystals with an irregular aggregate state.

**Figure 7:**
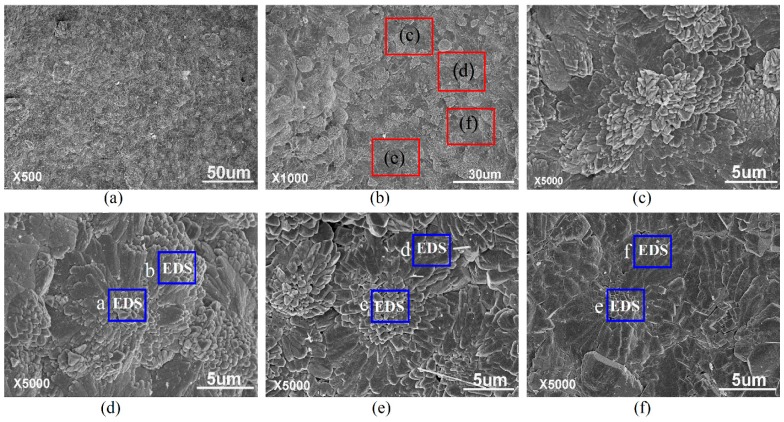
SEM images of GON/cement composite. (**a**) Magnified 500 times; (**b**) Magnified 1000 times; (**c**–**f**) Magnified 5000 times. The areas with blue markings in (**d**–**f**) are for energy-dispersive X-ray spectrometer (EDS) detection.

The cement composites mixed with GONs for the preparation of GON/cement composites. The SEM images of GON/cement composites at 28 days are shown in Figure 7. The results indicate that the entire cement matrix in GON/cement composites have an ordered microstructure that consists of polyhedron-like crystals with flower-like patterns. Figure 7a is a SEM image in a low magnification of 500 times, showing that the entire sample formed the ordered microstructure. Figure 7b is a SEM image in a magnification of 1000 times; from the image, it can be seen that that the ordered microstructure consists of polyhedron-like cement crystals via interweaving. Figure 7c–f shows four typical ordered microstructures with flower-like patterns, which assemble via the polyhedron hydration crystals. All cement hydration products became polyhedron-like crystals, and these polyhedron-like crystals assembled into ordered microstructures with flower-like patterns in the presence of GONs. These results suggest that there is a very capable organizer in the formation process of cement composites.

**Figure 8:**
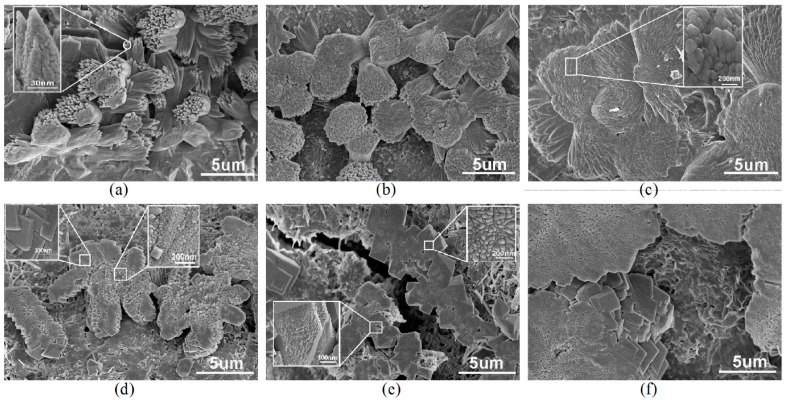
Formation and evolution process of cement hydration products controlled by GONs at 7 days: (**a**–**c**) Flower-like crystals; (**d**–**f**) Polyhedron-like crystals.

Generally, in the cement hydration process, producing some cracks and holes are inevitable. However, the above results show that GON/cement composites had dense and ordered microstructures with flower-like patterns at 28 days. In the research process, the microstructure of GON/cement composites in the initial stage of its formation was also investigated to reveal the regulation mechanism of GONs on the structure of cement composites. Figure 8 shows interesting SEM images of the composite at 7 days. The common feature of these SEM images is that the flower-like and polyhedron-like cement hydration products generate in the holes or the cracks of the composites. These results can help us to understand the formation process of cement hydration products under the control of GONs. Figure 8a shows that flower-like hydration products are easy to produce in the holes of cement composites [31]. Figure 8b shows that flower-like hydration products tend to form dense microstructures via growing aggregation. Figure 8c shows that flower-like products can form ordered microstructures with flower-like patterns and exhibit repairing effects for the holes and cracks. Therefore, Figure 8a–c exactly indicates the generating, growing, and forming process of the ordered microstructures of cement composites. Identically, Figure 8d–f shows that the polyhedron crystals are easy to produce in holes and cracks, and their growth can repair those holes and cracks. Figure 8 shows that cement hydration products have a repairing function for cracks and holes in a cement matrix.

**Figure 9:**
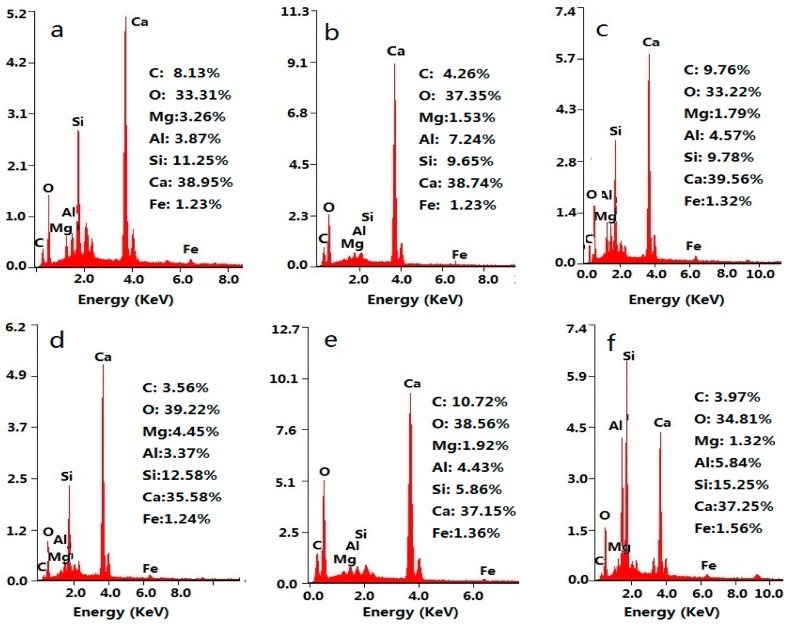
EDS results of the GON/cement composites in Figure 7d–f. (**a**) EDS spectra of a box in Figure 7d; (**b**) EDS spectra of b box in Figure 7d; (**c**) EDS sprctra of c box in Figure 7e; (**d**) EDS spertra of d box in the Figure 7e; (**e**) EDS spectra of e box in Figure 7e; (**f**) EDS spectra of box in Figure 7f.

In order to confirm the organizer of ordered microstructures, the chemical element compositions of the polyhedron aggregates with flower-like patterns were determined by EDS, and the results are shown in Figure 9. The EDS results indicate that the carbon content in the center of the flower-like patterns is relatively high compared with other places. This can act as evidence that GONs influence the shape and aggregate state of cement hydration products, especially in the center of the ordered microstructures with flower-like patterns. The results confirm GONs’ ability to regulate cement hydration products and microstructures. The ordered microstructures with a crosslinked network are beneficial for reducing cracks and improves the strength and toughness of cement composites.

The crystal structure of the cement hydration products of GON/cement composites was investigated via XRD spectra. Four randomly selected test samples were investigated, and the results are shown in Table 2. The results indicate that GONs have an important effect on the pore structure of GON/cement composites. GON/cement composites have a small total pore area, a median pore diameter, and an average pore diameter and porosity. The median pore diameter’s closeness to the average diameter indicated that the pore diameters were uniform. The results indicate that GONs can promote the formation of ordered microstructures with smaller, fewer cracks and holes. This explanation appears consistent with the SEM images of the GON/cement composites.

### 3.3. Strength and Durability of GON/Cement Composites

The compressive and flexural strength of GON/cement composites are shown in Table 3, from which it can be seen that GON/cement composites have made great improvements in compressive and especially flexural strength. The compressive and flexural strengths of GON/cement composites at 28 days increased by 51.1% and 85.1% compared with the control sample, respectively. The results are consistent with SEM morphology and the pore structure of GON/cement composites.

The microstructure of cement composites is very closely related to its durability. Properties closely related to durability, such as penetration resistance, freeze–thaw resistance, and carbonation resistance, were determined, and the results are shown in Table 4. The results indicate that durability parameters such as seepage height, freeze–thaw mass loss (***m*_loss_**), the retention rate of relatively dynamic elasticity modulus (***p***), and carbonation depth have markedly improved compared with the control samples. The results suggest, therefore, that GON/cement composites will have an improved service life.

### 3.4. Formation Mechanism of Regular Cement Hydration Products

**Figure 10:**
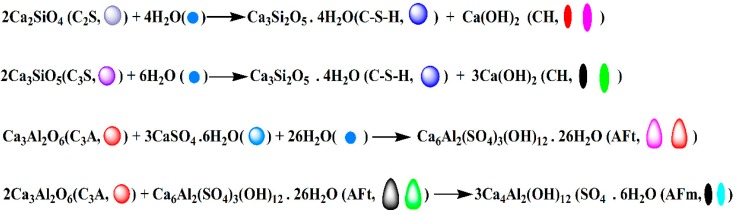
Schematic diagram of cement hydration reaction and products.

Cement mainly consists of tricalcium silicate C_3_S (Ca_3_SiO_5_), dicalcium silicate C_2_S (Ca_2_SiO_4_), tricalcium aluminate C_3_A (Ca_3_Al_2_O_6_), and a small amount of gypsum (CaSO_4_·2H_2_O). In the hydration process, C_3_A, C_3_S, and C_2_S will carry out a complex hydration reaction to form ettringite [Ca_6_Al_2_(SO_4_)_3_(OH)_12_·26H_2_O, AFt], monosulfate [Ca_4_Al_2_(OH)_2_·SO_4_·H_2_O, AFm], calcium hydroxide [Ca(OH)_2_, CH)], and calcium silicate hydrate [3CaO·2SiO_2_·4H_2_O, C–S–H] gel, the corresponding chemical reactions of which are represented by Figure 10. Generally, CH, Aft, and AFm exhibit rod-like and needle-like shapes with disorder. 

**Figure 11:**
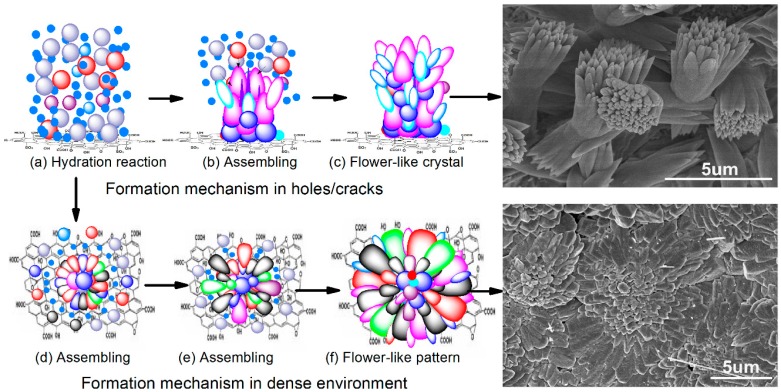
Schematic diagram of formation mechanism of order microstructure with flower-like patterns. (**a**) Hydration reaction; (**b**) Assembly effects of GONs; (**c**) Flower-like crystals; (**d**,**e**) Assembly effects of GONs; (**f**) Flower-like patterns.

According to the above results, a possible formation mechanism of GONs on the microstructures ofthe cement matrix is proposed, as shown in Figure 11. The surfaces of GONs have many chemical groups, such as –OH, –O–, and –COOH. These chemical groups react preferentially with C_3_S, C_2_S, and C_3_A when cement meets with water (Figure 11a). The initial products form growth points for the hydration products (Figure 11b,d), after which the hydration reaction continues as the formation of hydration products aggregate with flower-like patterns (Figure 11c,e). These flower-like crystals consist of AFt, AFm, CH, and C–S–H, and its shape is controlled by GONs. On aGON surface, a multitude of hydration crystals can interweave into a column of crystals growing from the GON surface. Once the column-shaped crystals grows into a pore, crack, or loose structure, they grow apart and form flower-like crystals, which disperse into pores and cracks, acting as fillers and crack arrestors (Figure 11a–c). When the cement hydration reaction has been in a dense environment, it produces a dense structure with a flower-like pattern and expands further (Figure 11d–f). Ordered microstructures with flower-like patterns can greatly contribute to improving strength [31,32].

## 4. Conclusions

(1) GONs can be used to control the formation of Portland cement hydration products into polyhedron-like crystals and an aggregate-forming ordered microstructure with flower-like patterns. The research results indicate that polyhedron products can transform into flower-like patterns and further form ordered microstructures with defect-free structures.
(2) These cement hydration products are easier to grow in cracks and holes of the cement matrix; therefore, they can repair structural defects through growth. The result is the formation of regular and interpenetrating networks via the crosslinking and growth of polyhedron-like cement hydration crystals. This ordered network is a new kind of microstructure in cement composites, which can significantly enhance the strength and toughness of cement.
(3) These results have major practical applications for the production of cement composites with high strength, high toughness, and long durability.

## Figures and Tables

**Table 1:** Chemical composition of the cement.

Component	SiO_2_	Al_2_O_3_	MgO	CaO	Na_2_O	K_2_O	SO_3_	Fe_2_O_3_	P_2_O_5_	TiO_2_	MnO
Content (%)	21.25	4.21	2.90	65.16	0.50	0.97	0.72	3.35	0.10	0.21	0.07

**Table 2:** Pore structure of GON/cement composites.

Specimens	Total Pore Area (m^2^/g)	Median Pore Diameter (nm)	Average Pore Diameter (nm)	Porosity (%)
Control sample	25.64	28.43	19.92	23.56
Sample 1	13.38	12.27	10.66	9.32
Sample 2	12.45	12.25	11.27	9.56
Sample 3	13.36	11.68	10.67	8.34
Sample 4	12.78	12.03	11.54	8.69

**Table 3:** Compressive and flexural strength of GON/cement composites.

Specimens	Compressive Strength (MPa)			Flexural Strength (MPa)		
	3 Days	7 Days	28 Days	3 Days	7 Days	28 Days
Control sample	26.52	45.67	57.42	3.18	6.84	8.33
GON/cement composites	35.65	62.56	86.75	4.72	12.37	15.42

**Table 4:** Durability testing results of GON/cement composites.

Specimens	Penetration Resistance		(Freeze Thaw Cycles * 100 Times)			Carbonation Depth (mm)	
	Osmotic Pressure (MPa)	Seepage Height (mm)	*m*_0_ (g)	*m*_loss_ (g)	*p* (%)	7 Days	28 Days
Control samples	3.5	13.6	9818	0.04	71.2	2.8	3.5
GON/cement composites	3.5	3.8	9860	0	93.6	0.6	1.2

* **m****_0_**: the weight of samples before freeze–thaw experiments; ***m*_loss_**: the weight of samples after 100 freeze–thaw cycles; ***p***: theretention rate of relatively dynamic elasticity modulus of the test samples after 100 freeze–thaw cycles.
